# Neoadjuvant stereotactic ablative body radiotherapy combined with surgical treatment for renal cell carcinoma and inferior vena cava tumor thrombus: a prospective pilot study

**DOI:** 10.1186/s12894-024-01405-y

**Published:** 2024-02-03

**Authors:** Jiyuan Chen, Zhuo Liu, Ran Peng, Yunchong Liu, Hongxian Zhang, Guoliang Wang, Xiaojun Tian, Xinlong Pei, Junjie Wang, Shudong Zhang, Hao Wang, Lulin Ma

**Affiliations:** 1 Department of Urology, Peking University Third Hospital, Beijing, 100191 China; 2 Department of Radiation Oncology, Peking University Third Hospital, Beijing, 100191 China; 3 Department of Radiology, Peking University Third Hospital, Beijing, 100191 China; 4 Cancer Center, Peking University Third Hospital, Beijing, 100191 China

**Keywords:** Renal cell carcinoma, Radiotherapy, Neoadjuvant therapy, Venous tumor thrombus

## Abstract

**Background:**

Surgical treatment for renal cell carcinoma (RCC) and inferior vena cava (IVC) tumor thrombus (TT) is difficult, and the postoperative complication rate is high. This study aimed to explore the safety and oncologic outcomes of neoadjuvant stereotactic ablative body radiotherapy (SABR) combined with surgical treatment for RCC and IVC-TT.

**Methods:**

Patients with RCC and IVC-TTs were enrolled in this study. All patients received neoadjuvant SABR focused on the IVC at a dose of 30 Gy in 5 fractions, followed by 2 ~ 4 weeks of rest. Then, radical nephrectomy and IVC tumor thrombectomy were performed for each patient. Adverse effects, perioperative outcomes, and long-term prognoses were recorded.

**Results:**

From June 2018 to January 2019, 8 patients were enrolled—4 with Mayo grade II TT and 4 with Mayo grade III TT. Four (50%) patients had complicated IVC wall invasion according to CT/MRI. All patients received neoadjuvant SABR as planned. Short-term local control was observed in all 8 patients. Only Grade 1–2 adverse events were reported. In total, 3 (37.5%) laparoscopic surgeries and 5 (62.5%) open surgeries were performed. The median operation time was 359 (IQR: 279–446) min, with a median intraoperative bleeding volume of 750 (IQR: 275–2175) ml. The median postoperative hospital stay was 7 (5–10) days. With a 26-month (range: 5–41) follow-up period, the estimated mean overall survival was 30.67 ± 5.38 months.

**Conclusions:**

This is the first preoperative radiotherapy study in Asia that focused on patients with TT. This study revealed the considerable safety of neoadjuvant SABR for RCC with IVC-TT.

**Trial registration:**

This study was registered in the Chinese Clinical Trials Registry on 2018-03-08 (ChiCTR1800015118). For more information, please see the direct link (https://www.chictr.org.cn/showproj.html?proj=25747).

**Supplementary Information:**

The online version contains supplementary material available at 10.1186/s12894-024-01405-y.

## Background

Renal cell carcinoma (RCC) is a malignant tumor of the urinary system that accounts for 3–5% of all adult cancers [1]. RCC is characterized by macrovascular invasion, which results in inferior vena cava (IVC) tumor thrombus (TT) in 4-10% of locally advanced RCC patients and is associated with progressive clinical symptoms and poor prognosis [2–4]. Surgical treatment, namely, radical nephrectomy and IVC tumor thrombectomy, is the standard treatment for RCC with IVC-TT. However, compared to RCC without IVC-TT, for RCC with IVC-TT, the surgical difficulty and postoperative complication rate is significantly increased, especially for complex cases, including those with high Mayo-level TT or TT with IVC wall invasion [5]. The latter is also associated with a greater possibility of IVC segmental resection and a higher risk of IVC-TT recurrence [4].

To decrease the surgical difficulty mentioned above, many attempts have been made to reduce IVC-TT complexity before surgical treatment, including through neoadjuvant target therapy and immunological therapy [6–8]. Recently, the resurgence of radiotherapy for the preoperative treatment of RCC has also been highlighted with the development of newer technologies. Stereotactic ablative body radiotherapy (SABR), which allows for high-dose delivery to focal lesions, was reported to have a high local control rate and favorable safety profile for primary tumors and RCC metastasis in clinical trials [9].

Therefore, we propose that SABR can be used as a neoadjuvant therapy for RCC with IVC-TT to potentially reduce surgical complexity by downgrading the IVC-TT and reversing IVC wall invasion. To date, few studies have reported the use of neoadjuvant SABR for IVC-TT treatment in RCC patients, and its safety and effectiveness have not been well demonstrated [10]. Here, we report the results from a prospective cohort of RCC patients with IVC-TTs who received neoadjuvant SABR and underwent surgical treatment to provide insights into the safety of neoadjuvant SABR for renal tumor thrombus and its effectiveness in decreasing surgical difficulty.

## Methods

### Patients

RCC patients diagnosed with Mayo grade II ~ IV IVC-TTs according to imaging examinations who were eligible for radical nephrectomy and IVC tumor thrombectomy were enrolled, as described in registered and published protocols (ChiCTR1800015118) [11]. The trial protocol and informed consent form were approved by the Peking University Biomedical Ethics Committee. Patients who could not tolerate the treatment or who had a previous history of systemic therapy or radiotherapy in the area of RCC or IVC-TT were excluded. This study was reported according to the CONSORT guidelines.

The overall preoperative evaluation included demographic information, clinical symptoms, physical examination data, and CT/MRI data. The IVC-TT stage and IVC wall invasion were re-evaluated after 2–4 weeks of rest following SABR treatment. Then, each patient underwent radical nephrectomy and IVC tumor thrombectomy, which were performed by two experienced urologic surgeons. Perioperative parameters, including the operative time, surgical bleeding, and postoperative complication rate, were recorded to assess surgical difficulty.

The primary endpoint of this study was safety, which was assessed by the occurrence of postoperative complications and SABR-related adverse events (AEs) 4–6 weeks after the radiotherapy course. The Clavien‒Dindo classification system was used to assess the severity of postoperative complications. SABR-related AEs were evaluated using the Common Terminology Criteria for Adverse Events (CTCAE) version 5.0. Severe toxicity was defined as Grade III-IV toxicity.

The follow-up time was defined as the time from the date of surgery to the latest documented date of telephone or clinical follow-up.

### Treatment plan

#### SABR

Only the IVC-TT was selected for the SABR treatment target. Target sections were delineated before SABR on the unenhanced CT phase. The IVC-TT was delineated according to the gross tumor volume (GTV), and the stomach, duodenum, jejunum, ileum, colon, spinal cord, liver, and esophagus were considered the organs at risk. The planning target volume (PTV) was generated by adding a 3-mm margin around the GTV. Patients were asked to lie on their backs with their hands at their sides to receive SABR. A total dose of 30 Gy in 5 fractions was delivered over 1 week. Further details are described in the published protocol [11].

#### Surgery

Open surgery and laparoscopic surgery were performed in this study, and the retroperitoneal approach is preferred for laparoscopy at our center. The patient position and trocar placement were described in previous studies [12, 13]. For right RCC with Mayo grade II IVC-TT, the right renal artery and right ureter were severed, and the distal end of the IVC, the left renal vein, and the proximal end of the IVC were dissected and blocked with vessel tourniquets successively. For right RCC with Mayo grade III IVC-TT, the hepatic ligament was severed to dissect the liver from the diaphragm, thus exposing and blocking the IVC under the diaphragm. The first hepatic portal was also dissected and blocked before IVC resection. For left RCC, the surgical process was similar to that for right RCC. However, the transperitoneal approach was applied for long and thick IVC-TTs, and the distal end of the IVC, right renal vein, and proximal end of the IVC were blocked. The right renal artery was also blocked if necessary. For patients with IVC wall invasion, IVC wall resection or segmental resection was used.

### Statistical analysis

The Shapiro‒Wilk (S‒W) test was used to determine the normality of continuous variables. Continuous variables with a normal distribution are presented as the mean ± standard deviation (SD); otherwise, they are presented as medians and interquartile ranges (IQRs). The Kaplan‒Meier method was used to calculate the overall survival rate.

## Results

From June 2018 to January 2019, a total of 8 RCC patients with Mayo grade II ~ III IVC-TTs were recruited for the study. The baseline characteristics are depicted in Table 1. Of the patients, 6 (75%) were male, and 2 (25%) were female. According to ECOG performance status, 2 (25%) patients received a score of 0, and 6 (75%) received a score of 1. Six patients had right-sided primary tumors, and 2 had left-sided tumors. Seven (87.5%) patients had grade cT1 tumors. One (12.5%) patient had a grade cM1 tumor. For the IVC-TT, 4 (50%) were Mayo grade II, and 4 (50%) were Mayo grade III. The average tumor thrombus length was 7.5 ± 3.6 cm. Four (50%) patients had complicated IVC wall invasion.

**Table 1:** Baseline characteristics of the patients who received neoadjuvant SABR

Variables	Case No.							
	1	2	3	4	5	6	7	8
Gender	Male	Female	Male	Male	Male	Female	Male	Male
Age	60	61	63	50	46	60	76	34
BMI	21.5	20.6	21.2	19.4	25.3	25.4	20.5	25.9
ECOG performance status	1	1	1	1	0	1	1	0
ASA classification	I	III	III	II	III	II	II	II
Tumor side	Right	Left	Right	Right	Right	Left	Right	Right
Tumor diameter/cm	10.4	5.6	7.5	8.3	12	5.6	14.2	12.2
Clinical T stage	3b	3b	4	4	3c	4	4	3b
Clinical N stage	1	1	1	1	1	1	1	0
Clinical M stage	0	0	0	0	0	0	1	0
Mayo grade	II	III	III	II	III	II	III	II
Tumor thrombus length/cm	4.6	5.5	8.9	3.5	14.4	5.7	10.1	6.5
Vascular wall invasion	No	No	No	Yes	Yes	Yes	Yes	No

BMI, body mass index; ECOG, Eastern Cooperative Oncology Group; ASA, American Society of Anesthesiologists

**Fig. 1:**
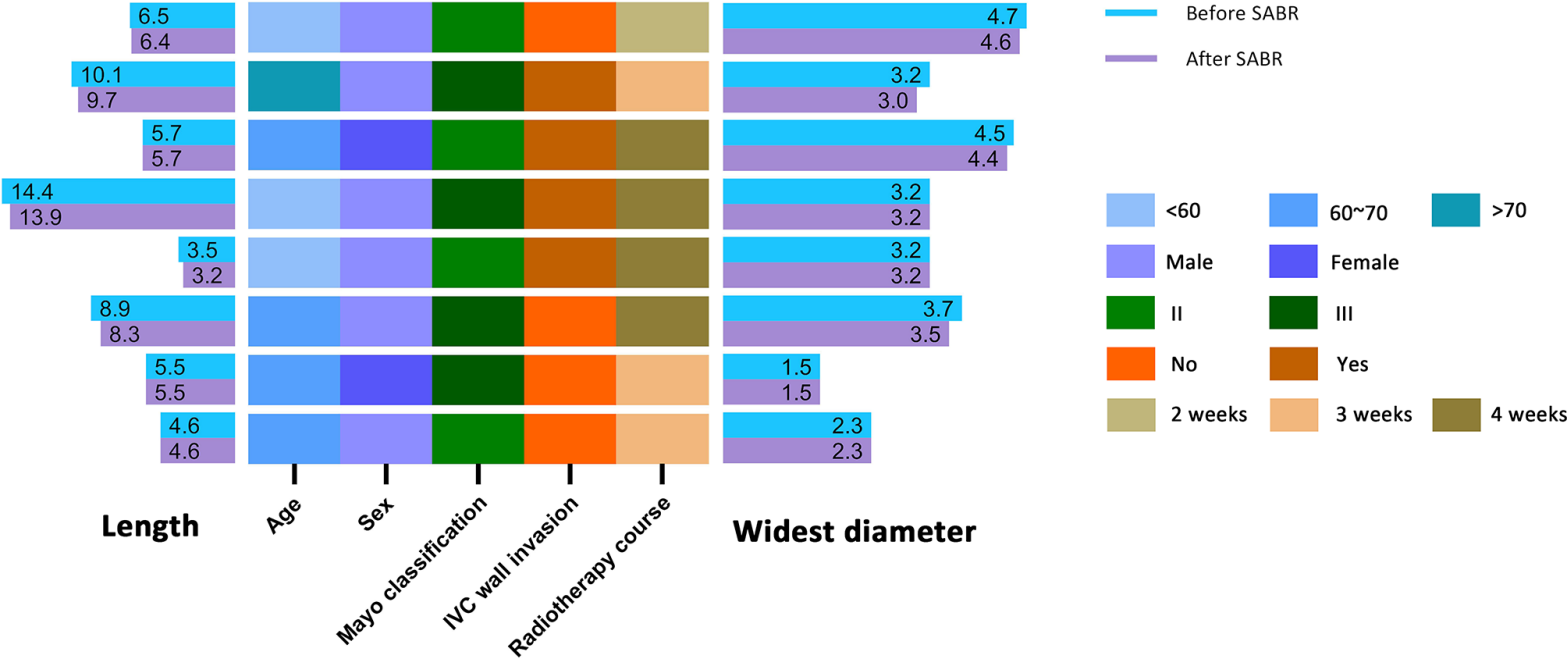
The clinical features of patients with IVC-TTs before and after neoadjuvant SABR. Left histogram, the length of IVC-TTs before and after SABR; heatmap, clinical profiles; right histogram, the widest diameter of IVC-TTs before and after SABR

**Fig. 2:**
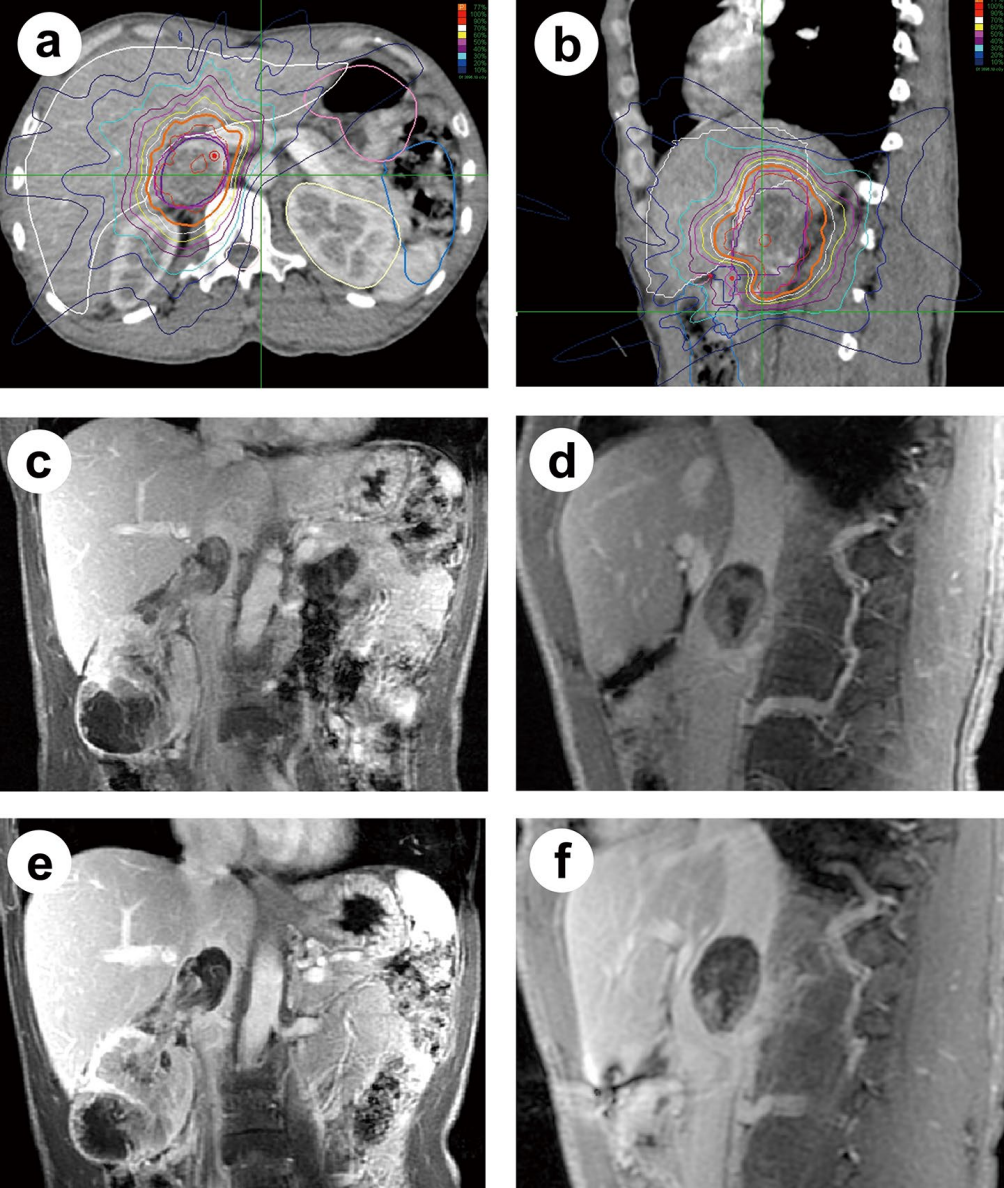
CT images showing the SABR treatment plan (**a**, **b**) and comparisons of IVC-TTs before (**c**, **d**) and after SABR (**e**, **f**). (**a**, **b**) The PTV of SABR on the cross-section and coronal planes. (**c**, **d**) Coronal views of the primary RCC tumor and Mayo grade II IVC-TT from a single patient before SABR. (**e**, **f**) CT images of the same primary tumor and IVC-TT after SABR, compared to those in c and d, respectively

Figure 1 shows the clinical features of the IVC-TTs before and after SABR treatment. Three (37.5%) patients received a 4-week course of radiotherapy, 4 (50%) patients received a 3-week course, and 1 (12.5%) patient received a 2-week course. The average tumor thrombus length after SABR was 7.2 ± 3.4 cm, which slightly reduced compared with baseline, and no new occurrence of IVC wall invasion was observed after SABR, therefore short-term local control was observed for all 8 patients. MRI showed decreased enhancement of IVC-TTs after SABR, which is consistent with the findings of previous case reports, probably because of local edema and vascular reduction in the IVC-TT (Fig. 2).

The perioperative results and SABR-related AEs are reported in Additional file 1. The operation was successful in all patients. Three patients (37.5%) underwent laparoscopic surgery, and 5 (62.5%) underwent open surgery. The median operation time was 359 (IQR: 279–446) min, with a median intraoperative bleeding volume of 750 (IQR: 275–2175) ml. The median postoperative hospital stay was 7 [5–10] days. No severe intraoperative adhesion or fragile tissue were observed in all patients. According to the Clavien‒Dindo classification system, level I postoperative complications included 1 case of hypokalemia, and level II complications included 3 cases of anemia and 1 case of lymphatic fistula. No severe postoperative complications (defined as Clavien‒Dindo classification ≥ III) were reported. Four to six weeks after the SABR treatment, no patient had anorexia, diarrhea, enterocolitis, gastritis, dermatitis radiation, bone marrow hypocellular, or hepatic failure. Only grade 1–2 AEs were reported.

**Fig. 3:**
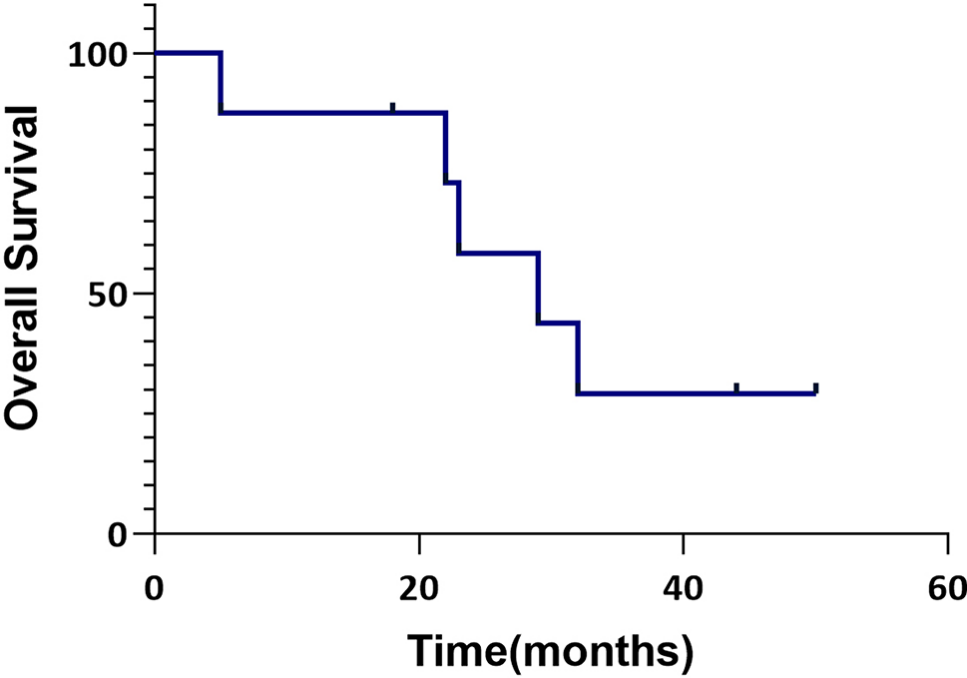
Kaplan‒Meier plot showing the overall survival of patients who received neoadjuvant SABR and underwent radical surgical treatment

The median follow-up time was 26 (range: 5–41) months, and the estimated mean overall survival was 30.67 ± 5.38 months. The Kaplan‒Meier curve was plotted to depict the overall survival curve, as shown in Fig. 3.

## Discussion

To the best of our knowledge, this is the first preoperative radiotherapy study in Asia that focused on TTs. This study aimed to determine the safety of neoadjuvant SABR for RCC IVC-TTs in terms of surgical complexity and adverse effects. 75% of the enrolled patients had Mayo grade III IVC-TTs or IVC wall invasion. Nevertheless, the surgical duration, intraoperative bleeding volume, and postoperative hospital stay were in line with our expectations when compared with previously reported experiences from our center [14]. In addition, there was no occurrence of severe postoperative complications or AEs; therefore, these patients met the primary endpoint of the study. The estimated mean survival also complied with the results presented previously for patients with IVC wall invasion.

To date, few studies have reported the application of SABR for RCC with IVC-TT, and most of the published studies are case reports and studies with a small sample size [10, 15–18]. The major studies are presented in Additional file 2. Three studies used SABR as an alternative therapy for surgery, and only one study focused on the safety of SABR as neoadjuvant therapy. Hannan et al. and Freifeld et al. were the earliest reporters to use SABRs for IVC-TT and metastasis treatment, with no occurrence of severe AEs [17, 18]. Additionally, one patient showed an excellent oncologic prognosis of 54 months. A larger cohort was reported by Freifeld et al. in 2022, in which a radiographic rate of 58% was reached and a significant symptom palliation effect was observed [16]. Recently, Castelnau-Marchand et al. reported a patient with IVC-TT and distant metastasis [15]. The patient received SABR and systemic therapy and achieved complete remission at the last follow-up of 54 months. Margulis et al. enrolled 6 patients who received neoadjuvant SABR (40 Gy in 5 fractions) and underwent radical nephrectomy and IVC thrombectomy after 4–14 days of rest [10]. No grade 4 or 5 AEs occurred, and all patients were alive after a median follow-up of 24 months. Exploratory analysis revealed decreased Ki-67 and increased PD-L1 expression in the IVC-TT, indicating activation of the immune system after SABR.

In comparison, our study enrolled more complex patients with higher IVC-TT grades, and half of them had IVC wall invasion. The results from these patients supported the conclusion of Margulis et al., indicating the safety of neoadjuvant SABR [10]. However, the radiographic response rate was not adequately reported in previous neoadjuvant studies and thus remains unclear. According to our results, no significant downgrading of the IVC-TT or reversal of IVC wall invasion was observed 2–4 weeks after SABR, but the radiographic results showed a decrease in enhancement, consistent with the observations of Freifeld et al. and Hannan et al., which probably resulted from local edema and vascular reduction in the IVC-TT [17, 18].

Few studies reported the adverse effect of neoadjuvant SABR on surgical difficulty. Although radiotherapy is associated with addition intraoperative adhesions and fragile tissues, no severe intraoperative adhesion or fragile tissue were observed in all patients. We suppose the absence of these adverse event resulted in the limited dosage and short SABR treatment interval, and the potential effect may require revaluation in higher dosage.

In theory, it is not easy to observe prominent changes in IVC-TT volume within a few weeks, especially during safety lead-in studies. Typically, several months are needed to observe a remarkable radiographic response, and a continual decrease in tumor volume can be observed within 1–2 years due to immune activation [19, 20]. In previous studies, although short-term changes were rarely reported, apparent reductions in IVC-TT size or even complete remission were recorded after several months, which indicated the potential effectiveness of SABR [15–18].

In addition, the growth rate of IVC-TTs is significantly faster than that of primary tumors, and even several-week intervals can lead to apparent progression [6, 21]. Froehner et al. reported a case of RCC with IVC-TT that progressed to Mayo grade III from Mayo grade I in one month [22]. In our study, although the IVC-TT volume did not decrease significantly within a few weeks, it remained stable or was slightly reduced. Therefore, we believe that these results can reveal the potential curative effect of SABR, as we used a relatively low dose of 30 Gy in 5 fractions. In the future, we will conduct a dose-escalation study to verify the effectiveness of neoadjuvant SABR for RCC patients with IVC-TTs.

Our study has several limitations. First, this was a single-arm cohort with a small sample size and some heterogeneity. Second, because of the patients’ strong demands for earlier surgery, the rest time following SABR was reduced to 2–4 weeks after providing informed consent, which is shorter than the planned period reported in the protocol. Thus, we recorded only the radiographic changes over a short-term interval. Finally, we enrolled only patients with Mayo grade II-III IVC-TTs, and further study is needed for Mayo grade IV IVC-TTs.

## Conclusions

This is the first preoperative radiotherapy study in Asia that focused on TT. Neoadjuvant SABR for RCC with IVC-TT has considerable safety and excellent oncologic outcomes. A further dose-escalation study, which we will be conducting, is needed to verify the potential effectiveness of this treatment.

### Electronic supplementary material

Below is the link to the electronic supplementary material.

**Supplementary Material 1:** Perioperative outcomes and AEs of patients who received radical nephrectomy and IVC thrombectomy after SABR

**Supplementary Material 2:** Published studies that reported SABR for RCC with IVC-TT

## Data Availability

All data are presented in the article and Supplementary Materials.

## Abbreviations

SABR: stereotactic ablative body radiotherapy
RCC: renal cell carcinoma
IVC: inferior vena cava
TT: tumor thrombus
AE: adverse effect
CTCAE: Common Terminology Criteria for Adverse Events
GTV: gross tumor volume
PTV: planning target volume
BMI: body mass index
ECOG: Eastern Cooperative Oncology Group
ASA: American Society of Anesthesiologists
OS: overall survival
