# Supplementary material for: Neoadjuvant stereotactic ablative body radiotherapy combined with surgical treatment for renal cell carcinoma and inferior vena cava tumor thrombus: a prospective pilot study
Source: BMC Urol. 2024 Feb 3;24:31. doi: 10.1186/s12894-024-01405-y (PMC10838433; doi:10.1186/s12894-024-01405-y)
Supplement: Supplementary file 1 — Supplementary Material 1: Perioperative outcomes and AEs of patients who received radical nephrectomy and IVC thrombectomy after SABR [file 12894_2024_1405_MOESM1_ESM.docx]

Additional file 2. Published studies that reported SABR for RCC with IVC-TT(8, 13-16).

| **Authors** | **Year of publication** | **Therapy type** | **Sample size** | **Tumor thrombus** | | | | **SABR** | | | | | | **Systematic therapy** | **Prognosis** | **(Refs.)** |
| --- | --- | --- | --- | --- | --- | --- | --- | --- | --- | --- | --- | --- | --- | --- | --- | --- |
|  |  |  |  | **Mayo classifications** | **IVC wall invasion** | **Thrombus diameters** | | **Irradiation site** | **PTV range** | **Dose-fractionation schedule for IVC-TT** | **Rest time for Neoadjuvant therapy** | **Curative effect** | **Adverse Effect** |  |  |  |
| Castelnau-Marchand et al. | 2023 | Alternative therapy for surgery | 1 | III | N/A | | N/A | IVC-TT and liver metastasis | GTV with a 3-mm margin | 35 Gy in 5 fractions | N/A | PET-CT showed no signs of IVC-TT in 4 months, 1 year, 36 months, 42 months and 54 months | No AE was reported | Nivolumab: IV q4wk | Complete remission at 54 months | 13 |
| Freifeld et al. | 2022 | Alternative therapy for surgery | 15 | I: 2 II: 5 III: 4 IV: 4 | N/A | | Median: 3 (1-4.1) cm | IVC-TT (100%) with/without metastasis (20%) | N/A | Median: 40 (25-50)/5 (1-5) | N/A | Radiographic response rate: 58% Symptom palliation was recorded in all patients receiving SABR for this indication | Only grade 1-2 AEs were reported | Before SABR: 11 (73.3%) After SABR: 8 (53.3%) | Median OS: 34 months | 14 |
| Margulis et al. | 2021 | Neoadjuvant therapy | 6 | I: 2 II: 4 | N/A | | N/A | IVC-TT | GTV with an additional 0.5 cm in the axial plane and 1.0 cm in the longitudinal plane | 40 Gy in 5 fractions | 4-14 days | Decreased Ki-67 and increased PD-L1 expression were observed in IVC-TT; Better host immune status were observed according to inflammatory cytokines and autoantibody titers. | grade 1: 73% (59/81) grade 2: 23% (19/81) grade3: 4% (3/81) | No until progression | All patients were alive after a median follow-up of 24 months； | 8 |
| Freifeld et al.; Hannan et al. | 2019; 2015 | Alternative therapy for surgery | 2 | IV: 2 | Case 1: Yes Case 2: N/A | | Case 1: N/A Case 2 : 4.9 cm | IVC-TT and metastasis | N/A | Case 1: 50 Gy in 5 fractions Case 2: 45 Gy in 5 fractions | N/A | Case 1: Reduction in size and enhancement of the IVF-TT Case 2: N/A | Case 1: No SAE was reported Case 2: N/A | Both discontinued after SABR | Case 1: The patient survived for 54 months since initial diagnosis, and 34 months after SABR, with no IVT-TT progress Case 2: The patients survived 18 months without systemic therapy | 15, 16 |

IVC = inferior vena cava; PTV = planning target volume; SABR = stereotactic ablative body radiotherapy; TT = tumor thrombus; AE = adverse event; OS = overall survival;
