# Supplementary material for: Neoadjuvant stereotactic ablative body radiotherapy combined with surgical treatment for renal cell carcinoma and inferior vena cava tumor thrombus: a prospective pilot study
Source: BMC Urol. 2024 Feb 3;24:31. doi: 10.1186/s12894-024-01405-y (PMC10838433; doi:10.1186/s12894-024-01405-y)
Supplement: Supplementary file 2 — Supplementary Material 2: Published studies that reported SABR for RCC with IVC-TT [file 12894_2024_1405_MOESM2_ESM.docx]

Addition file 1. Perioperative outcomes and AEs of patients who received radical nephrectomy and IVC thrombectomy after SABR.

| Variables | Patient No. | | | | | | | |
| --- | --- | --- | --- | --- | --- | --- | --- | --- |
|  | 1 | 2 | 3 | 4 | 5 | 6 | 7 | 8 |
| Operation approach | Laparoscopic | Laparoscopic | Open | Laparoscopic | Open | Open | Open | Open |
| Operation time/min | 358 | 359 | 431 | 451 | 320 | 540 | 245 | 265 |
| Intraoperative bleeding volume/ml | 200 | 50 | 2400 | 700 | 1500 | 4000 | 500 | 800 |
| Postoperative complications | Anemia | Lymphatic fistula | Anemia | - | Anemia | Hypokalemia | - | - |
| Postoperative hospital stay/days | 6 | 6 | 4 | 5 | 7 | 10 | 13 | 10 |
| Clevian-Dindo classification | 2 | 2 | 2 | - | 2 | 2 | - | - |
| Pathological N stage | 0 | 1 | 0 | 0 | 0 | 0 | 0 | 0 |
| Pathological type | Clear cell carcinoma | Clear cell carcinoma | Clear cell carcinoma | Clear cell carcinoma | Clear cell carcinoma | Clear cell carcinoma | Papillary renal cell carcinoma | Papillary renal cell carcinoma |
| AEs | Anemia | Nausea, vomiting, chylothorax | Nausea, fatigue, anemia | None | Anemia | Hypokalemia | Nausea, wound complication | None |

AE = Adverse event
